# Tarsal tunnel syndrome caused by an uncommon ossicle of the talus: A case report

**DOI:** 10.1097/MD.0000000000011008

**Published:** 2018-06-22

**Authors:** Chang Hwa Hong, Young Koo Lee, Sung Hun Won, Dhong Won Lee, Sang Il Moon, Woo Jong Kim

**Affiliations:** a Department of Orthopaedic Surgery, Soonchunhyang University Hospital Cheonan, Suncheonhyang 6-gil, Dongam-gu, Cheonan; b Department of Orthopaedic Surgery, Soonchunhyang University Hospital Bucheon, Jomaru-ro, Wonmi-gu, Bucheon; c Department of Orthopaedic Surgery, Soonchunhyang University Hospital Seoul, Daesagwan-ro, Yongsan-gu; d Department of Orthopaedic Surgery, Konkuk University Medical Center, Neungdong-ro, Gwangjin-gu, Seoul, Korea.

**Keywords:** os sustentaculi, tarsal tunnel syndrome, tibial nerve

## Abstract

**Rationale:**

Tarsal tunnel syndrome (TTS) is a compressive neuropathy of the posterior tibial nerve or one of its branches within the tarsal tunnel that is often caused by a variety of space-occupying lesions, such as ganglia, lipomas, varicosities, neural tumors, trauma, or systemic disease. The os sustentaculi is a small accessory bone, bridged to the posterior aspect of the sustentaculum tali by fibrocartilage. To the best of our knowledge, this is a rare case of successful treatment of TTS caused by the os sustantaculi.

**Patient concerns:**

A 37-year-old male presented with insidious onset of right ankle and foot pain for 1 year. He also complained of a tingling sensation and paresthesia from the plantar and medial aspect of the forefoot to the middle foot area along the main distribution of the medial plantar nerve. The symptoms were mild at rest, but increased upon prolonged walking. He had an ankle sprain history during a football game 2 years previously and recurrent ankle sprains had occurred more frequently in this ankle since that trauma.

**Diagnoses:**

Plain standing anteroposterior and lateral view radiographic findings of the right ankle reveled an accessory ossicle located posterosuperomedial to the sustentaculum tali. A computed tomography scan showed that the ossicle articulated between the talus and calcaneus. A magnetic resonance image revealed mild bone marrow edema in the ossicle and medial displacement of the tarsal structures.

**Interventions:**

Surgery was performed under general anesthesia. The ossicle was delineated from its surrounding structures and was removed. Tension on the nerve was released.

**Outcomes:**

The patient's pain and hypoesthesia were immediately relieved, and the tingling sensation disappeared 6 months after surgery. The patient had no complications or recurrence of symptoms at the 1-year follow-up.

## 1 Introduction

Tarsal tunnel syndrome (TTS) is a compressive neuropathy of the posterior tibial nerve or one of its branches within the tarsal tunnel that is often caused by a variety of space-occupying lesions, such as ganglia, lipomas, varicosities, neural tumors, trauma, or systemic disease.^[1–4]^ The os sustentaculi was first described by Ptitzner^[5]^ in 1896 as a small accessory bone, bridged to the posterior aspect of the sustentaculum tali by fibrocartilage. Of all accessory ossicles reported in the foot and ankle region, it constitutes a rare skeletal variant with an estimated incidence of 0.3% to 0.4%.^[6]^ We present a case of successful treatment of TTS caused by an os sustantaculi, which is rarely reported.

## 2 Case description

This case report was approved by the Institutional Review Board of Soonchunhyang University Hospital, and informed consent was received by patient. A 37-year-old male presented with insidious onset of right ankle and foot pain for 1 year. He also complained of a tingling sensation and paresthesia from the plantar and medial aspects of the forefoot to the middle foot area along the main distribution of the medial plantar nerve. The symptoms were mild at rest, but increased upon prolonged walking. He had an ankle sprain history during a football game 2 years previously, and recurrent ankle sprains were more frequent in the ankle since that trauma.

**Figure 1:**
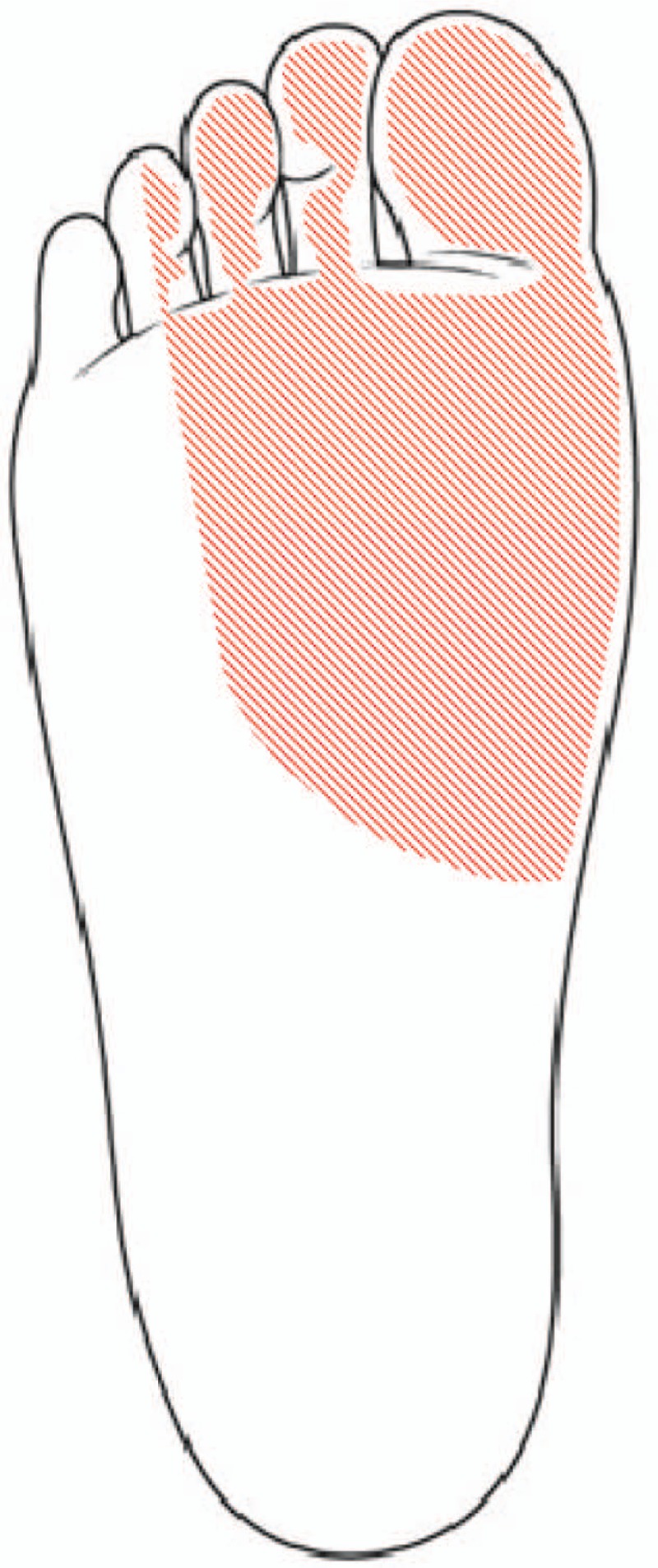
Distribution of hypoesthesia expressed by the patient.

**Figure 2:**
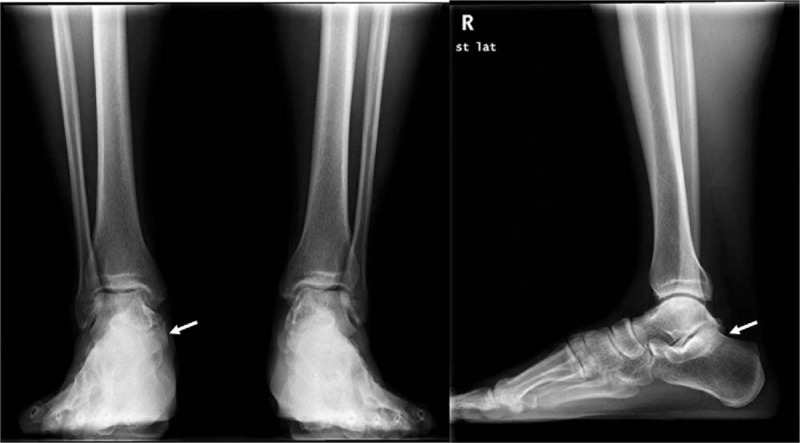
Preoperative plain standing anteroposterior (A) and lateral (B) radiographic views of the right ankle showing an accessory ossicle (arrow), which is located posterosuperomedially to the sustentaculum tali. The ossicle is the os sustentaculi.

**Figure 3:**
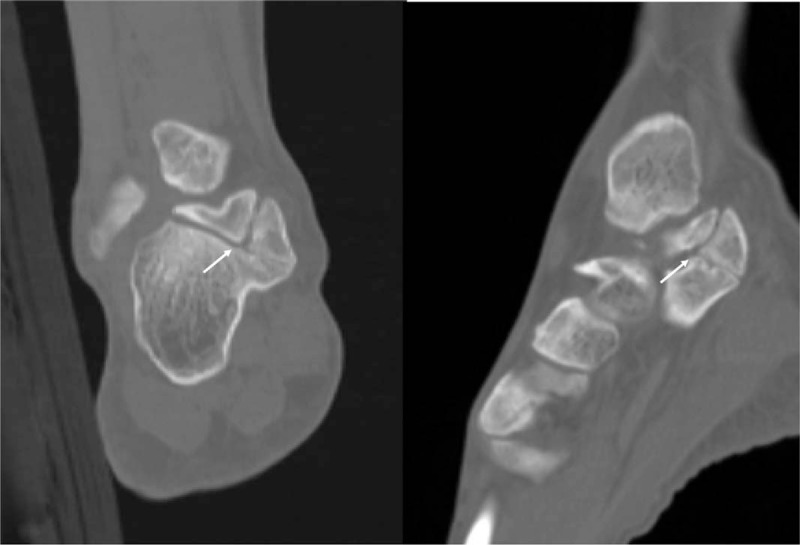
Computed tomography (CT) scan of the ossicle. Oblique coronal CT (A) and sagittal CT cuts showing the appearance of the ossicle and position of the accessory ossicle articulating between the talus and calcaneus. The accessory ossicle is united with the sustentaculum tali and a narrow and irregular interface (arrow) between the accessory ossicle and adjacent bones is noted. CT = computed tomography.

**Figure 4:**
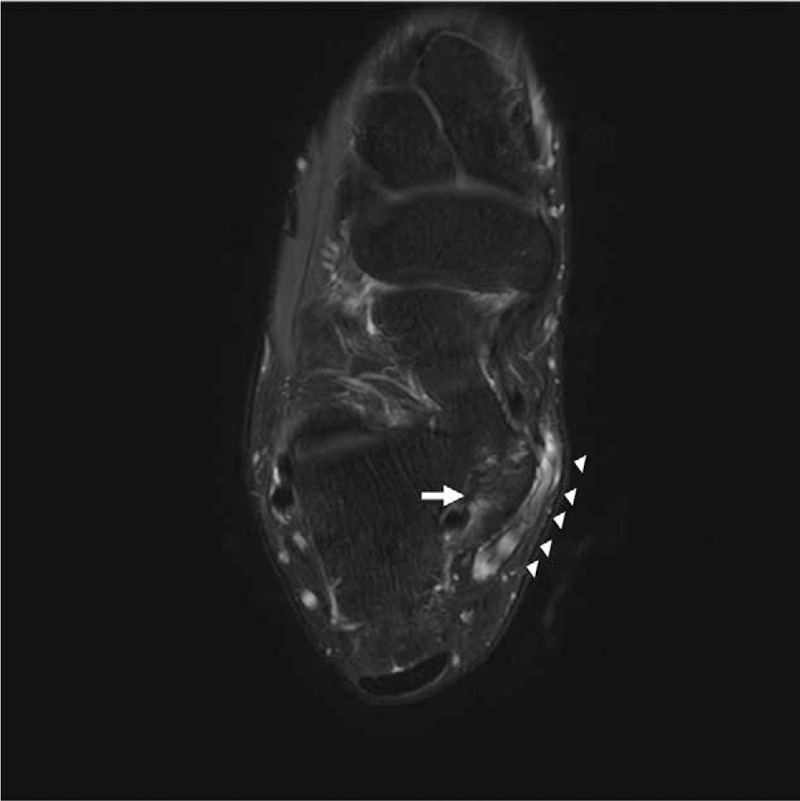
Preoperative axial, T2-weighted magnetic resonance image of the right ankle showing the accessory ossicle (arrow) in a close relationship with the sustentaculum tali. Mild bone marrow edema is noted in the ossicle, and the displaced medial tarsal structures are revealed (arrowheads).

A clinical examination showed a localized, bony, nontender, hard, mass-like swelling on the anteroinferior border of the medial malleolus. The patient stated said that he had been aware of the mass for 2 years. The posteroinferior side of the mass was tender, and Tinel's sign was positive. Hypoesthesia was present on the sore (Fig. 1). The plain radiographic findings of both standing anteroposterior and lateral views of the right ankle showed an accessory ossicle located posterosuperomedial to the sustentaculum tali (Fig. 2A and B). In addition, computed tomography (CT) and magnetic resonance imaging (MRI) were performed. Oblique coronal (Fig. 3A) and sagittal CT scans (Fig. 3B) showed the accessory ossicle articulating between the talus and calcaneus. The accessory ossicle united with the sustentaculum tali, and a narrow and irregular interface was noted between the accessory ossicle and adjacent bones. MRI showed that the accessory ossicle was closely associated with the sustentaculum tali. Mild bone marrow edema was noted in the ossicle and also revealed medial displacement of tarsal structures (Fig. 4). The American Orthopedic Foot and Ankle Society (AOFAS) Ankle-Hind Foot score was 45 points. Electromyography was not requested because the patient had presented with the classic signs and symptoms of TTS.

**Figure 5:**
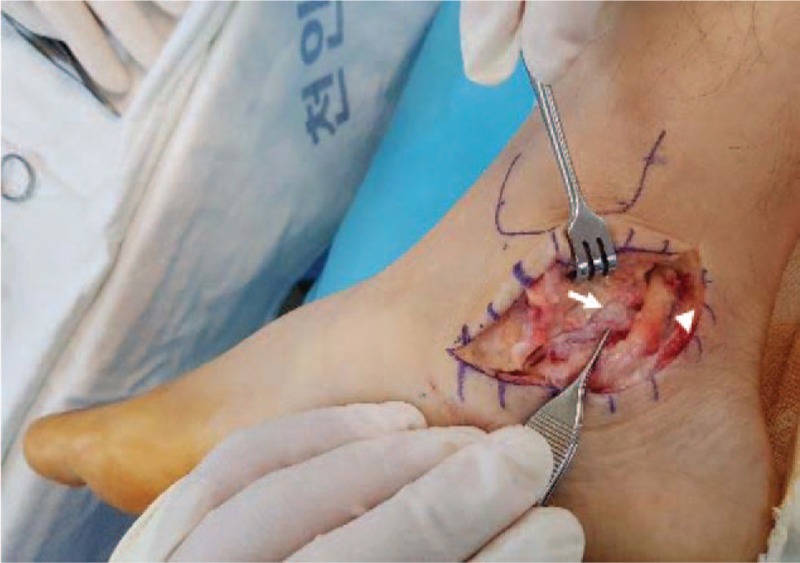
Intraoperative appearance of the ossicle (arrow) compressing the medial plantar nerve (arrowheads), which is a branch of the posterior tibial nerve.

Surgical decompression of the TTS was performed with the patient placed in the supine position with a pneumatic tourniquet under general anesthesia. Intraoperatively, the ossicle combined unstably with the fibrocartilaginous union on the posterosuperior border of sustentaculum tali, and the tibialis posterior nerve was compressed and stretched inward and downward by the ossicle when the flexor retinaculum was incised (Fig. 5). The tibialis posterior nerve and its branches were traced into the adductor halluces tunnel and released from their respective structures. The ossicle was delineated from its surrounding structures and was removed in full with an electric saw, osteotomes, and a rongeur, and the tension on the nerve was released. The flexor retinaculum was approximated with absorbable sutures, and the wound was closed in layers. The wound healed uneventfully during the follow-up period, and a rebound ankle brace was applied to the ankle for 6 weeks. After the surgery, the patient's pain and hypoesthesia were immediately relieved, and the tingling sensation disappeared 6 months after surgery. He had no complications or recurrence of symptoms at the 1-year follow-up. The AOFAS Ankle-Hind Foot score improved to 90 points. He had no complications or recurrence of symptoms at the 1-year follow-up.

## 3 Discussion

TSS is a compressive entrapment neuropathy of the posterior tibial nerve or one of its branches over the tarsal tunnel, posterior to the medial malleolus. The tarsal tunnel is a fibro-osseous tunnel that is bound by the flexor retinaculum superficially, the medial surface of the talus, the sustentaculum tali, the medial calcaneal wall laterally, and the abductor halluces muscle inferiorly.^[7]^ It is often caused by a variety of space-occupying lesions such as ganglia, lipomas, varicosities, neural tumors, trauma, or systemic disease.^[1–4]^

Supportive history and physical exam are the essential part in the diagnosis of TSS.^[8]^ Diagnostic triads of TSS are: pain and paresthesia in the distribution of the posterior tibial nerve branches a positive Tinel's sign, and positive electrodiagnostic studies.^[9]^ No single test can be used to diagnose TSS.^[9]^ Electrodiagnostic studies are helpful to support the diagnosis in 80%.^[1]^ Plain radiographs and additional US scan or MRI are helpful to exclude bony abnormalities and space-occupying lesions.^[10]^

The os sustentaculi is an accessory bone connected by fibrous tissue or fibrocartilage to the posterior aspect of the sustentaculum tali. The os sustentaculi is located medially and slightly dorsal (superior) to the sustentaclulum tali.^[11–13]^ It is mostly asymptomatic but can cause symptoms in some patients.^[6,11,14]^ The symptoms are caused by osteoarthritic changes or shearing stress forces of synchondrosis between the talus and calcaneus, but they are not definite.^[14,15]^

Although os sustentaculi is not a common cause of TTS, the large ossicle, which projects to the sustentaculum tali, can compress the posterior tibial nerve or one of its branches and cause TTS.

## 4 Conclusion

TTS is a common disease seen in foot and ankle orthopedic clinics with multiple etiologies. A thorough account of one's medical history, precise clinical examination, radiographs, and nerve conduction studies are mandatory to reveal the cause of TTS. After diagnosis and preoperative planning, proper treatment should be given to patients to improve outcomes.

## Acknowledgments

The authors would like to thank the Soonchunhyang University Research Fund for support.

## Author contributions

**Investigation:** Dhong Won Lee, Sang Il Moon.

**Supervision:** Chang Hwa Hong, Young Koo Lee.

**Writing – original draft:** Woo Jong Kim.

**Writing – review & editing:** Sung Hun Won.
